# Detecting Ethambutol Resistance in *Mycobacterium tuberculosis* Isolates in China: A Comparison Between Phenotypic Drug Susceptibility Testing Methods and DNA Sequencing of *embAB*

**DOI:** 10.3389/fmicb.2020.00781

**Published:** 2020-05-08

**Authors:** Ma-chao Li, Rong Chen, Shi-qiang Lin, Yao Lu, Hai-can Liu, Gui-lian Li, Zhi-guang Liu, Xiu-qin Zhao, Li-li Zhao, Kang-Lin Wan

**Affiliations:** ^1^State Key Laboratory for Infectious Disease Prevention and Control, Collaborative Innovation Center for Diagnosis and Treatment of Infectious Diseases, National Institute for Communicable Disease Control and Prevention, Chinese Center for Disease Control and Prevention, Beijing, China; ^2^Pathogenic Biology Institute, University of South China, Hengyang, China; ^3^Department of Bioinformatics, College of Life Sciences, Fujian Agriculture and Forestry University, Fuzhou, China; ^4^School of Laboratory Medicine and Life Sciences, Wenzhou Medical University, Wenzhou, China

**Keywords:** *Mycobacterium tuberculosis*, ethambutol resistance, phenotypic drug susceptibility testing, DNA sequencing, microplate alamarBlue assay

## Abstract

With the increasing incidence of drug-resistant tuberculosis (DR-TB), determining a rapid and accurate drug susceptibility testing (DST) method to identify ethambutol (EMB) resistance in *Mycobacterium tuberculosis* has become essential for patient management in China. Herein, we evaluated the correlation between three phenotypic DST methods, namely, proportion method (PM), MGIT 960 system, and microplate alamar Blue assay (MABA), and DNA sequencing of *embAB* in 118 *M. tuberculosis* isolates from China. When the results of the phenotypic DST methods were compared with those of DNA sequencing, the overall agreement and kappa values of the PM, MGIT 960 system, and MABA were 81.4% and 0.61, 77.1% and 0.55, and 84.7% and 0.67, respectively. The agreement for EMB resistance between MABA and PM was significantly higher than that between the MGIT 960 system and PM (*P* = 0.02). Moreover, among the isolates with detectable *embAB* mutations, 97.2% (70/72 isolates) harbored mutations in *embB*. The analysis of *embB* mutations predicted EMB resistance with 81.3% sensitivity, 86.8% specificity, and 83.1% accuracy. Thus, MABA may be a better phenotypic DST method for detecting EMB resistance. DNA sequencing of *embB* may be useful for the early identification of EMB resistance and the consequent optimization of the treatment regimen.

## Introduction

Despite a decreasing trend in the incidence of tuberculosis (TB) in recent years, TB remains a major public health threat in China; in 2018, the World Health Organization (WHO) estimated 900,000 new cases and 37,000 deaths (World Health Organization [WHO], 2018). According to the latest national baseline survey, 5.7% of new cases and 26.5% of previously treated cases are drug-resistant TB (DR-TB) (Zhao et al., 2012). DR-TB, particularly multidrug-resistant TB (MDR-TB), generally requires lengthy, more costly, and more toxic treatment regimens, which are associated with higher risks of treatment failure and disease relapse. Rapid and accurate detection of drug resistance is thus crucial for timely optimizing treatment regimens and reducing treatment failure to prevent the transmission of DR-TB and the development of MDR-TB.

Ethambutol (EMB) is an essential first-line anti-TB drug that is routinely recommended in combination with other drugs for treating TB and DR-TB. It also serves as a key drug in second-line regimens for MDR-TB when drug susceptibility testing (DST) results are available. EMB acts against bacterium through inhibiting membrane-associated arabinosyl transferases, encoded by the *embCAB* operon (*embC*, *embA*, and *embB*) (Telenti et al., 1997; Safi et al., 2013), which are necessary for biosynthesis of arabinogalactan in the cell wall. Previous studies showed that the majority of EMB-resistant isolates carry mutations within *embB*, primarily at codon 306 (*embB*306) (Zhang et al., 2013; Brossier et al., 2015), suggesting that the mutations might be used as promising diagnostic markers for the rapid detection of EMB resistance. Furthermore, certain mutations within the upstream region (UR) of *embA* are associated with EMB resistance (Cui et al., 2014; Brossier et al., 2015; Zhao et al., 2015). However, the presence of these mutations in *embAB* was also observed in EMB-susceptible isolates (Shi et al., 2011; Zhao et al., 2015; Li et al., 2016; Sun et al., 2018). Besides, there are no mutations within the particular genes responsible for EMB resistance in some EMB-resistant isolates, implying the involvement of other resistance mechanisms (Sharma and Bisht, 2017; Sharma et al., 2018). As per the literature, significant discordance exists between the phenotypic and genotypic methods for EMB susceptibility testing (Kim, 2005; Garrigo et al., 2007; Cheng et al., 2014; Zhang et al., 2014). Hence, it is necessary to evaluate concordance across phenotypic and genotypic methods for EMB resistance testing. In the present study, we compared the correlation between three phenotypic EMB susceptibility testing methods and DNA sequencing of *embAB* mutations in 118 *Mycobacterium tuberculosis* (*M. tuberculosis*) isolates from China.

## Materials and Methods

### *M. tuberculosis* Isolates and DST

In total, 118 *M. tuberculosis* isolates collected from 118 unrelated patients with pulmonary TB were contained in this study. DST of TB isolates to antitubercular drugs isoniazid (INH), rifampin (RIF), streptomycin (SM), and EMB was performed on Lowenstein–Jensen (L-J) proportion method (PM) according to the WHO guideline, with the critical concentrations being 0.2 μg/ml for INH, 40 μg/ml for RIF, 4.0 μg/ml for SM, and 2.0 μg/ml for EMB (World Health Organization [WHO], 2009). EMB susceptibility testing was also performed with all isolates using the MGIT 960 system and microplate alamarBlue assay (MABA). The MGIT 960 system was carried out according to the manufacturer’s instructions, with the critical concentration of 5.0 μg/ml (Garrigo et al., 2007). MABA was performed as previously described (Leonard et al., 2008). Briefly, the isolate in the log phase was adjusted with saline to McFarland standard 1 and diluted 1:25 in 7H9 broth containing 10% OADC (BD, Franklin Lakes, NJ, United States). Then, 100 μl bacterial suspension was used to inoculate each well of the 96-well plate. EMB was serially diluted twofold in 100 μl 7H9 broth, and the final concentrations were 32, 16, 8, 4, 2, 1, and 0.5 μg/ml. The final volume of each well was 200 μl, containing 100 μl of EMB solution and 100 μl of bacterial suspension. Furthermore, EMB-free (inoculum-only) controls were used to determine the addition time for alamarBlue. Plates were sealed and incubated at 37°C for 1 week. The indicator (25 μl alamarBlue mixed with 25 μl 10% Tween-80) was added to the EMB-containing group when the drug-free control showed a color change (blue to pink). The minimum inhibitory concentration (MIC) value was defined as the lowest EMB concentration that inhibited bacterial growth and prevented a color change. The breakpoint MIC for EMB was taken as 4 μg/ml (Leonard et al., 2008).

### DNA Amplification and Sequencing

The hot regions conferring EMB resistance, including *embA* UR (145 bp upstream to codon 164) and *embB* (codons 159–518), of all isolates were amplified using the following primers: *emb-*S1, AACCTAGGAACGGTGACT, and *emb-*A1, CAACCTGTGGCTTCTTCT; *emb-S*2, AACT TCGTCGGGCTCAAG, and *emb-A*2, TAACGCAGGTTC TCGGTATA (Sun et al., 2018). The PCR program included an initial denaturation at 94°C for 5 min, followed by 30 cycles of denaturation at 94°C for 40 s, annealing at 58°C for 40 s, and extension at 72°C for 1 min and a final extension at 72°C for 5 min. All amplified products were then sequenced. The sequence data were aligned to the H37Rv reference genome (GenBank accession number NC_000962) using BioEdit 7.05.3.

### Data Analysis

Data were analyzed with SPSS 16.0 statistical software (SPSS Inc., Chicago, IL, United States). Statistical analysis was performed using the chi-square test or Fisher’s exact test as appropriate, and probability levels < 0.05 were considered as statistically significant. Agreement between different testing results was evaluated with the kappa statistic. The kappa values of < 0.2, 0.2–0.4, 0.41–0.6, 0.61–0.8, and 0.81–1.0 indicated poor agreement, fair agreement, moderate agreement, good agreement, and excellent agreement, respectively (Altman, 1999).

## Results

Based on DST by PM, 105, 99, 69, and 72 isolates were resistant to INH, RIF, SM, and EMB, respectively. Overall, 92 isolates were MDR, and 47 isolates were resistant to all four first-line drugs. Eight isolates were poly-drug resistant while five isolates were sensitive to all four drugs.

Of the 118 isolates, 72, 55, and 80 were identified to be EMB resistant by the PM, MGIT 960 system, and MABA, respectively (Table 1). For the 72 isolates determined as being EMB resistant by the PM, 61 (84.7%, 61/72 isolates) harbored at least one mutation within *embAB*. By contrast, 11 (23.9%) of 46 phenotypically susceptible isolates harbored *embAB* mutations. Further, 50 (90.1%) of the 55 isolates identified to be EMB resistant by the MGIT 960 system showed *embAB* mutations, with the mutations also occurring in 22 of 63 (34.9%) EMB-susceptible isolates. Finally, 67 (83.8%) of the 80 isolates determined to be EMB resistant by MABA carried *embAB* mutations; five (13.2%) of 38 EMB-susceptible isolates also showed *embAB* mutations. When results of the three phenotypic DST methods were compared with those of DNA sequencing results, the overall agreement and kappa values were 81.4% and 0.61, 77.1% and 0.55, and 84.7% and 0.67 for the PM, MGIT 960 system, and MABA, respectively. Table 1 summarizes the results of MABA in comparison with those of the PM and MGIT 960 system. The concordance rate for EMB resistance between MABA and PM was significantly higher than that between MGIT 960 and PM (*P* = 0.02), with overall agreement/kappa values of 89.8%/0.78 and 78.8%/0.59, respectively.

**TABLE 1:** Agreement between phenotype DST and DNA sequencing.

DST method results	No. of isolates with sequencing results				No. of isolates with MABA results			
		
	M	NM	Agreement (%)	Kappa value	R	S	Agreement (%)	Kappa value
**PM**								
R	61	11	81.4	0.61	70	2	89.8	0.78
S	11	35			10	36		
**MGIT 960 system**								
R	50	5	77.1	0.55	55	0	78.8	0.59
S	22	41			25	38		
**MABA**								
R	67	13	84.7	0.67	/	/	/	/
S	5	33			/	/		

*Abbreviations: M, mutation; NM, no mutation; R, resistant; S, susceptible.*

All isolates displaying discordant results between the MGIT 960 system/PM and MABA were further analyzed (Table 2). Twenty-five isolates were determined to be susceptible by the MGIT 960 system but resistant by MABA, including 17 isolates (68.0%, 17/25 isolates) with *embAB* mutations. Of them, 18 (72.0%, 18/25 isolates) had MICs of 4 μg/ml, four (16.0%, 4/25 isolates) had MICs of 8 μg/ml, and three (12.0%, 3/25 isolates) had MICs of 16 μg/ml. In addition, two isolates without *embAB* mutations were determined to be resistant by PM but susceptible by MABA, and they had MICs of 1 μg/ml. In contrast, 10 isolates, including six isolates (60.0%, 6/10 isolates) harboring *embAB* mutations, were identified to be susceptible by PM but resistant by MABA; of them, seven isolates (70.0%, 7/10 isolates) had MICs of 4 μg/ml, and three isolates (30.0%, 3/10 isolates) had MICs of 16 μg/ml.

**TABLE 2:** Analysis of isolates with discordant results between the PM/MGIT 960 system and MABA.

Resistance patterns obtained during DST with			Sequencing results	No. of isolates

PM	MGIT 960 system	MABA (MICs μg/ml)		
R	S	S (1.0)	NM	2
S	S	R (4.0)	NM	2
S	S	R (4.0)	M	5
R	S	R (4.0)	M	10
R	S	R (4.0)	NM	1
R	S	R (8.0)	NM	3
R	S	R (8.0)	M	1
S	S	R (16.0)	NM	2
S	S	R (16.0)	M	1

*Abbreviations: R, resistant; S, susceptible; M, mutation; NM, no mutation.*

Of the isolates with detectable *embAB* mutations, 97.2% (70/72 isolates) had mutations within *embB* (Table 3). All *embB* mutations existed within codons 306–497. An amino acid replacement at codon 306 was the most frequent and occurred in 58 isolates (82.9%, 58/70 isolates). Met306 was noted to be replaced by Val (38 isolates), Ile (15 isolates), and Leu (five isolates). Of all the isolates with the *embB*306 mutation, three showed MICs of < 4 μg/ml (0.5, 1.0, and 2.0 μg/ml). The next most prevalent mutated codon was 406. Mutations at this codon were observed in seven isolates, two of which had MICs lower than 4 μg/ml (2 μg/ml). Other mutations were detected at codons 319 (three isolates), 328 (two isolates), 497 (two isolates), and 412 (one isolate). In addition, 14 EMB-resistant isolates displayed nucleotide changes within *embA* UR, including at −43 (two isolates), −16 (five isolates), −12 (three isolates), −11 (three isolates), and −5 bp sites (one isolate). Notably, most mutations within *embA* UR were accompanied by other mutations in *embB.* Only two isolates carried single *embA* UR mutations at −43 and −11 bp, with MICs of 8 μg/ml.

**TABLE 3:** Mutations in *embCAB* among 118 *M. tuberculosis* isolates.

Mutations		No. of isolates						
	
*embA *UR**	*embB*	MICs (μ g/ml)						
		
		≤0.5	1.0	2.0	4.0	8.0	16.0	≥32.0
G(-43)C		0	0	0	0	1	0	0
C(-11)T		0	0	0	0	1	0	0
C(-16)G	Met306Ile	0	0	0	0	0	1	0
C(-16)T	Met306Ile	0	0	0	0	1	0	1
C(-16)A	Met306Ile	0	0	0	0	1	0	0
	Met306Ile/Gly406Asp	0	0	0	0	0	1	0
	Met306Ile/Gly406Ser	0	0	0	0	1	0	0
	Met306Ile	1	0	0	4	2	2	0
G(-5)A	Met306Leu	0	0	0	0	1	0	0
	Met306Leu	0	1	0	0	1	2	0
G(-43)C	Met306Val	0	0	0	0	0	0	1
C(-16)G	Met306Val	0	0	0	0	0	1	0
C(-12)T	Met306Val	0	0	0	0	1	0	1
C(-11)A	Met306Val	0	0	0	0	0	0	2
	Met306Val/Gln497His	0	0	0	0	0	0	1
	Met306Val	0	0	1	6	18	3	3
	Tyr319Cys	0	0	0	0	2	1	0
	Asp328Tyr	0	0	0	1	0	1	0
C(-12)T	Gly406Ala	0	0	0	0	0	0	1
	Gly406Ala	0	0	0	0	1	1	0
	Gly406Asp	0	0	2	0	0	0	0
	Ser412Pro	0	0	0	0	0	0	1
	Gln497Arg	0	0	0	0	0	1	0
NM	NM	20	8	5	8	3	1	1

*Abbreviation: NM, no mutation.*

With reference to the results of MABA, the sensitivities, specificities, and accuracies for detection of the EMB resistance via DNA sequencing analysis of the different regions and codons in *embAB* are summarized in Table 4. Screening *embB* achieved satisfactory accuracy (83.1%). Inclusion of *embA* increased the assay accuracy to 84.7%. In addition, analyzing the *embB*306 mutations predicted EMB resistance with 68.8% sensitivity, 92.1% specificity, and 76.3% accuracy.

**TABLE 4:** Summary of DNA sequencing of *embAB* and MABA.

Locus or codon	No. of isolates				*P*-value	Sensitivity (%)	Specificity (%)	Accuracy (%)
	
	Resistant		Susceptible					
		
	M	NM	M	NM				
*embA*	14	66	0	38	0.02*	17.5	100.0	44.1
*embA*(-16)	5	75	0	38	0.28	6.3	100.0	36.4
*embB*	65	15	5	33	0.00**	81.3	86.8	83.1
*emb306*	55	25	3	35	0.00**	68.8	92.1	76.3
*embAB*	67	13	5	33	0.00**	83.8	86.8	84.7

Abbreviations: M, mutation; NM, no mutation. *P < 0.05 (significant); **P < 0.01 (highly significant).

## Discussion

In China, phenotypic DST based on L-J solid media and the liquid culture system MGIT 960 is routinely performed with all *M. tuberculosis* isolates. The presence of *embAB* mutations, particularly *embB*306, which are believed to be the major cause of EMB resistance in *M. tuberculosis*, has also been reported in EMB-susceptible isolates (Plinke et al., 2009; Zhao et al., 2015; Li et al., 2016; Sun et al., 2018). Because these routine phenotypic DST methods are notoriously problematic (Garrigo et al., 2007; Cheng et al., 2014; Zhang et al., 2014), presumably inaccurate results may be the major cause for the detection of “EMB-susceptible” isolates harboring *embAB* mutations (Plinke et al., 2009; Campbell et al., 2011). Identifying false susceptibility to EMB is of considerable importance for the successful treatment of DR-TB, especially MDR-TB, as treatment regimens for these patients should include any active first-line drug for improved outcomes. Thus, there is increasing emphasis on developing a reliable method to detect EMB resistance.

In this study, we found that in comparison with DNA sequencing, the overall agreement values for the PM, MGIT 960 system, and MABA were 81.4, 77.1, and 84.7%, respectively. The kappa values were 0.61 (good agreement), 0.55 (moderate agreement), and 0.67 (good agreement) for these three phenotypic DST methods. As evident, EMB resistance determined by MABA showed the best correlation with DNA sequencing results. We also examined concordance across all three phenotype DST methods by calculating the kappa coefficients. There was a good agreement for EMB between PM and MABA (κ, 0.78). Although PM is the gold standard recommended by the WHO and the current standard method used in China for EMB susceptibility testing of *M. tuberculosis* isolates, it requires at least 28 days before results can be obtained. As a rapid phenotypic DST based on liquid media, MABA can reduce the turnaround time to less than 14 days, which makes it a better choice for identifying EMB resistance.

Twenty-seven isolates exhibited discordant results between the PM/MGIT 960 system and MABA. Of them, 18 isolates (66.7%) had MICs of 4.0 μg/ml, which was the breakpoint MIC definition of EMB resistance. Furthermore, there were four (14.8%) isolates with MICs of 8.0 μg/ml, which is close to the breakpoint MIC. EMB is a slow-acting anti-TB drug, and culture-based methods can be problematic because of the bacteriostatic nature of EMB. The storage conditions of the drug and the type of medium used also influence the drug activity (Piersimoni et al., 2006). The MIC range of EMB-susceptible and EMB-resistant strains was relatively narrow (Madison et al., 2002; Cheng et al., 2014), and the critical breakpoints of different phenotypic DST methods were different (Madison et al., 2002; Banu et al., 2014; Cheng et al., 2014). Additionally, the presence of microcolonies on solid media and the proximity of EMB MICs of clinical isolates near the critical concentration could make determination of resistance difficult (Parsons et al., 2004; Plinke et al., 2009). Significant discrepancies were encountered when comparing the results obtained in different phenotypic DST methods (Plinke et al., 2009; Cheng et al., 2014; Ahmad et al., 2016). Hence, there were reports suggesting that molecular tests for detecting EMB resistance warranted accurate EMB susceptibility results (Lacoma et al., 2015; Ahmad et al., 2016).

With reference to results of MABA, 67 (83.8%) of 80 EMB-resistant isolates contained at least one mutation in *embAB*. This result validated that *embAB* mutations are associated with EMB resistance (Brossier et al., 2015; Zhao et al., 2015). The majority of *embAB* mutations were concentrated in *embB*. Of them, *embB*306 was the most frequent and was detected in 68.8% (55/80 isolates) isolates. The *embB*306 mutation was also detected in EMB-susceptible isolates. However, the mutation frequency in EMB-resistant isolates 68.8% (55/80 isolates) was significantly higher than that in EMB-susceptible isolates (7.9%, 3/38 isolates). A previous study indicated these mutations were likely to be important, in that they represent the first step toward acquiring EMB resistance (Safi et al., 2013), indicating the need to gain insight into the molecular basis of EMB resistance. It is also desirable to have a better understanding of treatment outcomes and treatment failure in patients infected with strains carrying *embB* mutations. Despite the fact that 14 EMB-resistant isolates harbored *embA* mutations in this study, 12 had additional mutations in *embB*. These multiple mutations were shown to be correlated with high-level EMB resistance (Sun et al., 2018).

Although 83.8% EMB-resistant isolates were identified with DNA sequencing of *embAB*, the inclusion of *embA* in molecular diagnoses only marginally increased the testing sensitivity by 2.5%. The best strategy at present for molecular diagnostics is selective targeting of *embB* (81.3% sensitivity, 86.8% specificity, and 83.1% accuracy). Analyzing the *embB*306 mutation could predict EMB resistance with 68.8% sensitivity, 92.1% specificity, and 76.3% accuracy. Thus, the effects of this mutation on detecting EMB resistance were relatively limited.

There were still some EMB-resistant isolates determined by phenotypic DST methods (PM, MGIT, and MABA), which lacked *embAB* mutations. This implied that these strains probably harbored mutations outside *embAB* (Safi et al., 2013; He et al., 2015; Tulyaprawat et al., 2019) or that the resistance may have been caused by other mechanisms, such as overexpressed efflux pumps and loss of porins (Sharma and Bisht, 2017; Sharma et al., 2018). Careful interpretation of a negative DNA sequencing result (no mutation) is thus necessary in the accurate management of suspected DR-TB.

This study also contained several limitations. (i) The number of isolates included was relatively limited. Additional research containing more isolates will be required to evaluate the effectiveness of the MABA and DNA sequencing. (ii) DNA sequencing is unable to identify EMB-resistant isolates that do not carry mutations in *embAB*. (iii) The association between *embB*306 mutation and other drug resistance was not evaluated.

## Conclusion

Our results indicated that MABA is suitable for detecting EMB resistance, showing good concordance with traditional PM methods and *embAB* mutations. DNA sequencing to screen *embB* may be useful for the rapid identification of EMB resistance. However, we do not suggest phenotypic DST methods to be replaced with DNA sequencing in China. It may be used as an initial diagnosis tool for the early identification of EMB resistance and consequently optimizing the treatment regimens.

## Data Availability Statement

The raw data supporting the conclusions of this article will be made available by the authors, without undue reservation, to any qualified researcher.

## Ethics Statement

The studies involving human participants were reviewed and approved by the Ethics Committee of National Institute for Communicable Disease Control and Prevention, Chinese Center for Disease Control and Prevention. The patients/participants provided their written informed consent to participate in this study.

## Author Contributions

LZ and K-LW conceived and designed the study. LZ, ML, HL, and SL analyzed the data. ML, RC, YL, GL, ZL, and XZ performed the laboratory experiments. ML, RC, YL, and HL contributed to the data collection and analysis. LZ, K-LW, and SL wrote the manuscript. All authors read and approved the final manuscript.

## Conflict of Interest

The authors declare that the research was conducted in the absence of any commercial or financial relationships that could be construed as a potential conflict of interest.
